# Effect of the “Art Coloring” Online Coloring Game on Subjective Well-Being Increase and Anxiety Reduction During the COVID-19 Pandemic: Development and Evaluation

**DOI:** 10.2196/37026

**Published:** 2022-07-08

**Authors:** JuZhe Xi, YuHan Gao, Na Lyu, Zhuang She, XinYue Wang, Xin-An Zhang, XiaoYu Yu, WeiDong Ji, MengSheng Wei, WeiHui Dai, Xuesheng Qian

**Affiliations:** 1 Shanghai Key Laboratory of Mental Health and Psychological Crisis Intervention Affiliated Mental Health Center (ECNU), School of Psychology and Cognitive Science East China Normal University Shanghai China; 2 Xinhua Hospital Shanghai Jiao Tong University of Medicine Shanghai China; 3 College of Letters and Science University of California Berkeley, CA United States; 4 Antai College of Economics & Management Shanghai Jiao Tong University Shanghai China; 5 School of Management Shanghai University Shanghai China; 6 Affiliated Mental Health Center (ECNU) Shanghai Changning Mental Health Center Shanghai China; 7 School of Management Fudan University Shanghai China

**Keywords:** coloring game, online intervention, mental health, COVID-19 pandemic, gamification, game-based intervention, commercially released game

## Abstract

**Background:**

COVID-19 has spread worldwide and generated tremendous stress on human beings. Unfortunately, it is often hard for distressed individuals to access mental health services under conditions of restricted movement or even lockdown.

**Objective:**

The study first aims to develop an online digital intervention package based on a commercially released coloring game. The second aim is to test the effectiveness of difference intervention packages for players to increase subjective well-being (SWB) and reduce anxiety during the pandemic.

**Methods:**

An evidence-based coloring intervention package was developed and uploaded to an online coloring game covering almost 1.5 million players worldwide in January 2021. Players worldwide participated to color either 4 rounds of images characterized by awe, pink, nature, and blue or 4 rounds of irrelevant images. Participants' SWB and anxiety and the perceived effectiveness of the game in reducing anxiety (subjective effectiveness [SE]) were assessed 1 week before the intervention (T1), after the participants completed pictures in each round (T2-T5), and after the intervention (T6). Independent 2-tailed *t* tests were conducted to examine the general intervention (GI) effect and the intervention effect of each round. Univariate analysis was used to examine whether these outcome variables were influenced by the number of rounds completed.

**Results:**

In total, 1390 players worldwide responded and completed at least 1 assessment. Overall, the GI group showed a statistical significantly greater increase in SWB than the general control (GC) group (N=164, t162=3.59, Cohen d=0.59, 95% CI 0.36-1.24, *P*<.001). Compared to the control group, the best effectiveness of the intervention group was seen in the awe round, in which the increase in SWB was significant (N=171, t169=2.51, Cohen d=0.39, 95% CI 0.10-0.82, *P*=.01), and players who colored all 4 pictures had nearly significant improvements in SWB (N=171, F4,170=2.34, partial ŋ2=0.053, *P*=.06) and a significant decrease in anxiety (N=171, F4,170=3.39, partial ŋ2=0.075, *P*=.01).

**Conclusions:**

These data indicate the effectiveness of online psychological interventions, such as coloring games, for mental health in the specific period. They also show the feasibility of applying existing commercial games embedded with scientific psychological interventions that can fill the gap in mental crises and services for a wider group of people during the pandemic. The results would inspire innovations to prevent the psychological problems caused by public emergencies and encourage more games, especially the most popular ones, to take more positive action for the common crises of humankind.

## Introduction

COVID-19 has been spreading rapidly, with 237,890,892 confirmed cases and 4,852,328 deaths worldwide as of October 2021 [1]. The COVID-19 pandemic has threatened people’s physical health as well as psychological health. Distress, depression, anxiety, posttraumatic stress symptoms, and insomnia are prevalent among people suffering from the pandemic [2,3]. Although emergency measures, such as social distancing and lockdown, helped prevent the spread of COVID-19, they also reduced people’s access to social support and mental health services. Thus, accessing psychological aid became critical during the pandemic.

Numerous interventions have been developed to improve people’s mental health during the COVID-19 crisis. These interventions typically use counseling services or self-guided programs for specific populations, including patients with COVID-19, health care workers, and people with mental illness [4,5]. However, the demerits of these interventions include limited popularization and a focus on narrow segments of people affected by the pandemic. Given the high prevalence of psychological distress (eg, anxiety, stress), new intervention models are needed to make mental health services accessible to the general population.

A promising solution is online self-guided interventions, which have the advantages of overcoming spatial barriers and being accessible to a broad population. For example, participants in Sweden who completed a 3-week online intervention reported significantly lower scores of worry [6]. However, the usefulness of such online interventions is largely restricted in experiments as most of them are not engaging enough. For the general population, it is often hard to adhere to such intervention programs due to limited interest in them.

We turn to digital games to address this issue. Digital games are entertaining in nature and have the potential to be designed into an attractive and effective intervention. For example, a recent study showed that virtual reality–based digital video games are effective in the treatment of arachnophobia [7]. This suggests that digital games have potential value as interventions to improve mental health. However, no game feature so far has been effective in improving mental health without degrading the game experience during the pandemic, especially commercially released games.

Coloring has been proved to reduce psychological distress and improve mood [8-10]. Coloring therapy is based on the idea that coloring can help people get away from their negative “inner dialogue” and negative thoughts and emotions [11]. This therapy has drawn wide research attention because coloring books have become popular among adults to relieve stress [12,13]. A study in Taiwan found that older adults who completed mandala coloring reported significantly lower anxiety levels and higher levels of calm, safety, and satisfaction than those in the control group [9]. In addition, online coloring games are currently popular (eg, Art Coloring & Color by Number, April Coloring, Lake Coloring), with a great number of fans worldwide. This alternative to digital coloring books has lower barriers to participation, more accessible operation, and more flexible cross-regional delivery. Although in the report, 1 limitation of physical coloring games is that unfinished coloring may cause additional psychological burdens [8], user-friendly online coloring can greatly simplify the coloring process and avoid it. Another limitation is that the improvement may be short term [8], but it also could be used as an accessible intervention for the general population during this particular period.

Four elements have been widely shown to be helpful in previous empirical studies [14-25]. First, coloring that elicits awe can reduce anxiety and strengthen happiness. Awe may transfer people’s attention from themselves to a bigger picture and increase positive feelings, such as connectedness and humility, which benefit people, especially when facing adverse events [14,15]. Second, coloring that provides a sense of being in nature, such as viewing nature pictures or connecting with the natural environment, can improve mood and reduce anxiety [16,17]. Contact with nature may help people recover from stressful situations and improve attention concentration, subsequently enhancing mental health [18,19]. Third, the color pink is positively related to warmth, love, and nurturance, effectively reducing stress and violent behaviors within even minutes of exposure [20-23]. Fourth, the color blue, a cold color with a short wavelength, has calming and soothing effects and the function to benefit mood and reduce depression and anxiety [23-25]. The effectiveness of awe, pink, nature, and blue in reducing psychological distress suggests that coloring images characterized by these 4 elements online could be useful in reducing distress and improving psychological well-being during the COVID-19 pandemic.

In this study, we developed an online coloring package based on the commercially released Art Coloring app to address mental health issues during the pandemic. The advantage is that the coloring game can include a large number of players across regions, who can directly access the intervention and avoid the entry and learning threshold of a new game. We implemented the coloring package using 4 evidence-based image types (awe, pink, nature, and blue). We tested the effectiveness of the online coloring game in increasing subjective well-being (SWB) and reducing anxiety. We hypothesized that the digital gamification mental intervention would be effective in the pandemic; in addition, participants who colored specific types of images would show higher levels of improvement in SWB and lower levels of anxiety than participants who colored other images.

## Methods

### Ethical Considerations

The study was approved by East China Normal University’s research ethics committee (HR151-2020).

### Procedure and Participants

**Figure 1:**
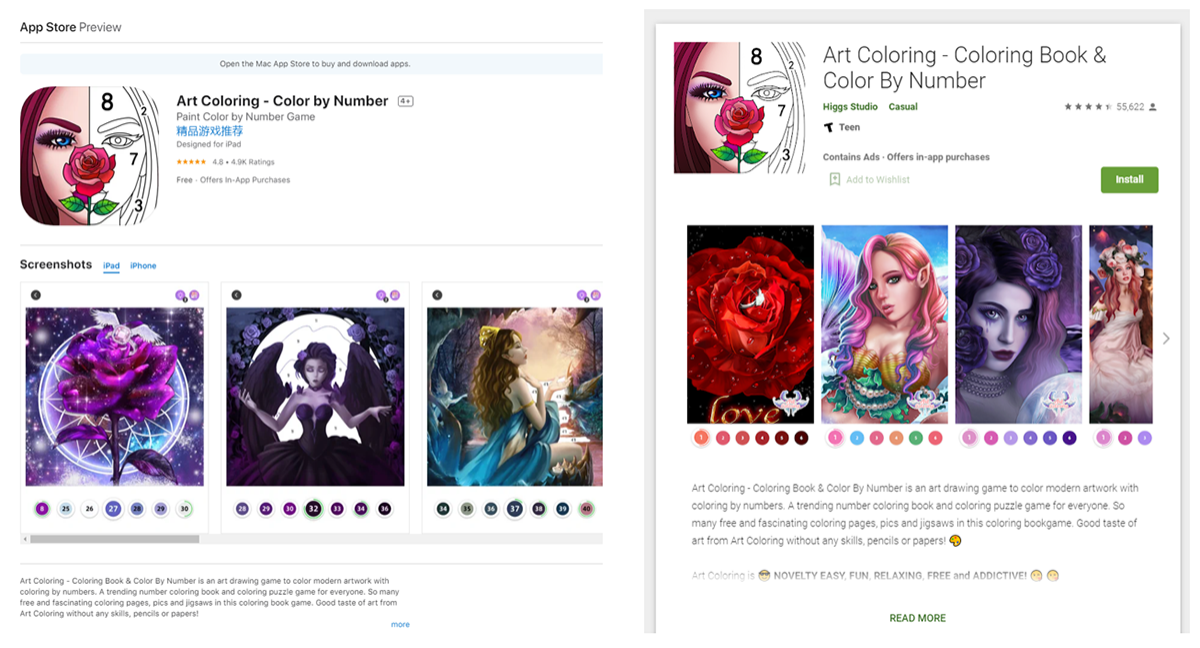
“Art Coloring - Coloring Book & Color by Number” in Apple App Store.

A commercially released app-based art drawing game called *Art Coloring - Coloring Book & Color by Number*, developed by Shiyi Network (Boke Technology Co., Ltd Company), was used as the intervention. The game has 1.5 million downloads, with over 80,000 daily active users (DAU) worldwide. Players worldwide can download the game on mobile phones or iPads from Google Play Store or Apple App Store and color line pictures following a series of numbered blocks to create colored pictures (Figure 1).

The participants in this study were existing players of the game. In addition to the basic coloring function, the game functions mainly involved in the study were “Daily Push” and “Daily News.” Daily Push automatically sent all users 8 basic pictures daily, and Daily News published game notices nonscheduled.

**Figure 2:**
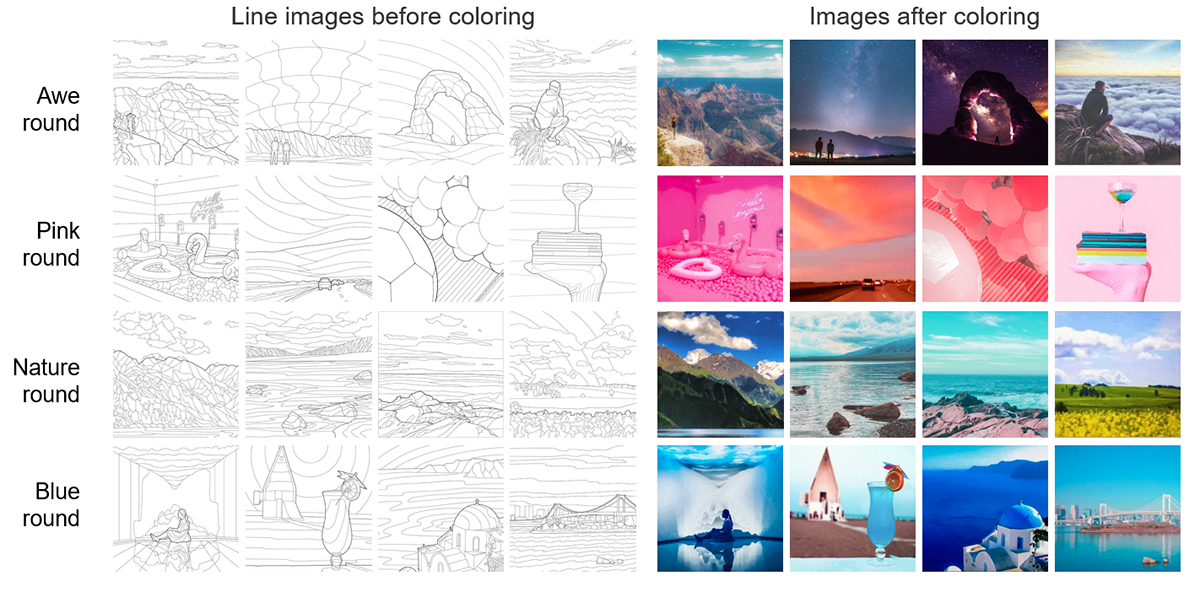
Coloring pictures for the awe, pink, nature, and blue rounds.

**Figure 3:**
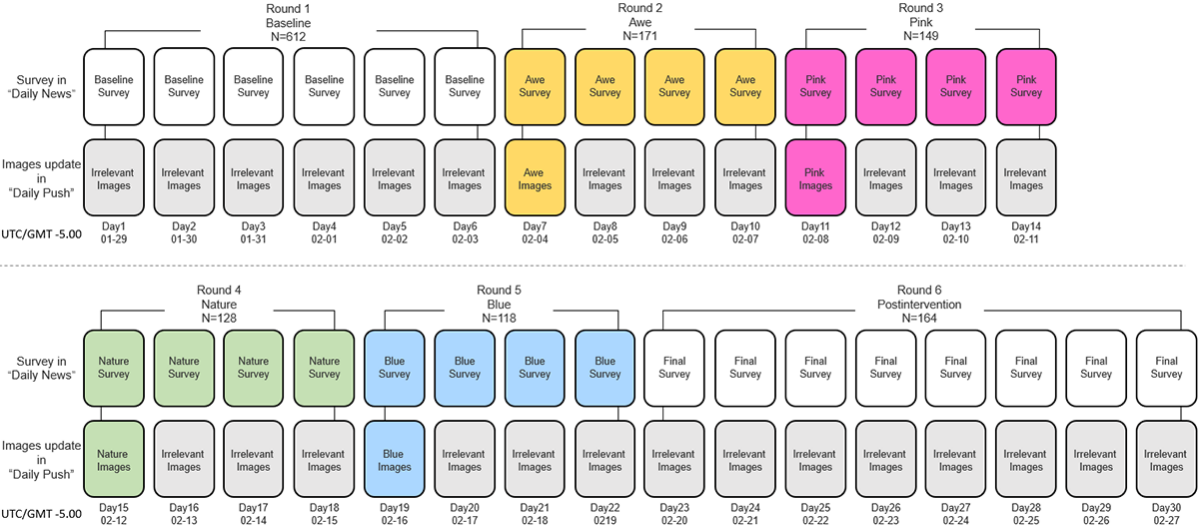
The study procedure.

In the study, Daily Push sent the 4 intervention coloring pictures mixed with 4 irrelevant pictures to players on schedule, following the group round order of awe, pink, nature, and blue (Figure 2); 1 group of pictures for 4 days was defined as 1 round of coloring. Every day, all players were free to choose and color the intervention images or irrelevant images (that do not have any of the 4 elements) and provide feedback through the Polls for Pages questionnaire embedded in Daily News. The procedure is shown in Figure 3. The questionnaire encouraged participants to leave email addresses and promised to draw 20 participants among all recorded emails to reward them with US $20 Amazon gift cards. The email addresses would be used as match codes, and cookies were placed on the players' devices to avoid duplicate submissions.

As shown in Figure 3, the study was conducted from January 29 to February 27, 2021. Every day for 16 days, all players were sent 1 group of coloring images. The participants’ SWB and anxiety and the perceived effectiveness of the game in reducing anxiety (subjective effectiveness [SE]) of the participants were measured immediately after each round and right after the intervention. Self-report questionnaires were administered at baseline (T1), after each round of coloring was completed (T2-T5), and right after the intervention (T6). The questionnaire administrated after the intervention (T6) aimed to collect data from more participants, especially those who did not complete the last-round questionnaire. The methodology ensured that, in addition to questionnaires, all players’ gaming experience would be the same as usual, which is the goal of this study—achieve an unnoticeable psychological enhancement when dealing with the psychological threat of a major crisis.

Two types of assessment were conducted to assess the intervention's effectiveness, both based on repeated measures. The general intervention (GI) effect was calculated based on the completed baseline and postintervention assessment of SWB, anxiety, and SE. The effect of each round of the intervention was calculated based on the assessment after each round.

Sociodemographic characteristics were collected at baseline, including the participants’ gender, age, country, and average time spent on the game per day.

Outcome variables were SWB, anxiety, and SE. Each outcome was measured with 1 item to ease the participants’ burden. SWB was measured using the Face Scale, a widely used single-item tool on which the participant rates their SWB by choosing 1 of 7 faces [26]. Anxiety was measured using the question “How have you been these 2 days?” Players rated their emotional state from 1 (very anxious) to 8 (very relaxed). The item was reverse-scored, with higher scores suggesting a higher level of anxiety. The SE was measured using the question “How effective do you think art coloring is in decreasing your anxiety level in general?” Players were asked to rate the item from 1 (no effect) to 8 (very effective).

### Statistical Analysis

To control for baseline scores, residual postintervention scores were calculated. In addition, the intervention and control groups were compared to examine the intervention effect of each category of images. The number and group of players in each round varied depending on whether the player chose to color the image assigned for that round, to color unrelated images, or to not do any coloring. For those who only completed 1 postround questionnaire after the baseline assessment, residual scores were calculated as the difference between postround scores and baseline scores. Residual scores of those who completed the prior round were calculated as the difference between the current postround and prior postround scores. The total number of intervention package pictures that the participants colored was coded for analysis as follows: 0 (did not color any experimental pictures), 1 (colored 1-4 pictures), 2 (colored 5-8 pictures), 3 (colored 9-12 pictures), and 4 (colored 13-16 pictures).

Statistical analysis was conducted using SPSS Statistics 21.0 (IBM Corporation). Independent 2-tailed *t* tests were conducted to compare the intervention and control groups on the residual scores for the dependent variables (SWB, anxiety, and SE). Independent 2-tailed *t* tests and chi-square tests were conducted to investigate differences in independent variables between groups. Univariate analysis was performed to examine the association between the number of completed rounds and outcome variables. Covariates were entered to control for any baseline differences between the groups in the analyses.

## Results

### Participant Characteristics

**Figure 4:**
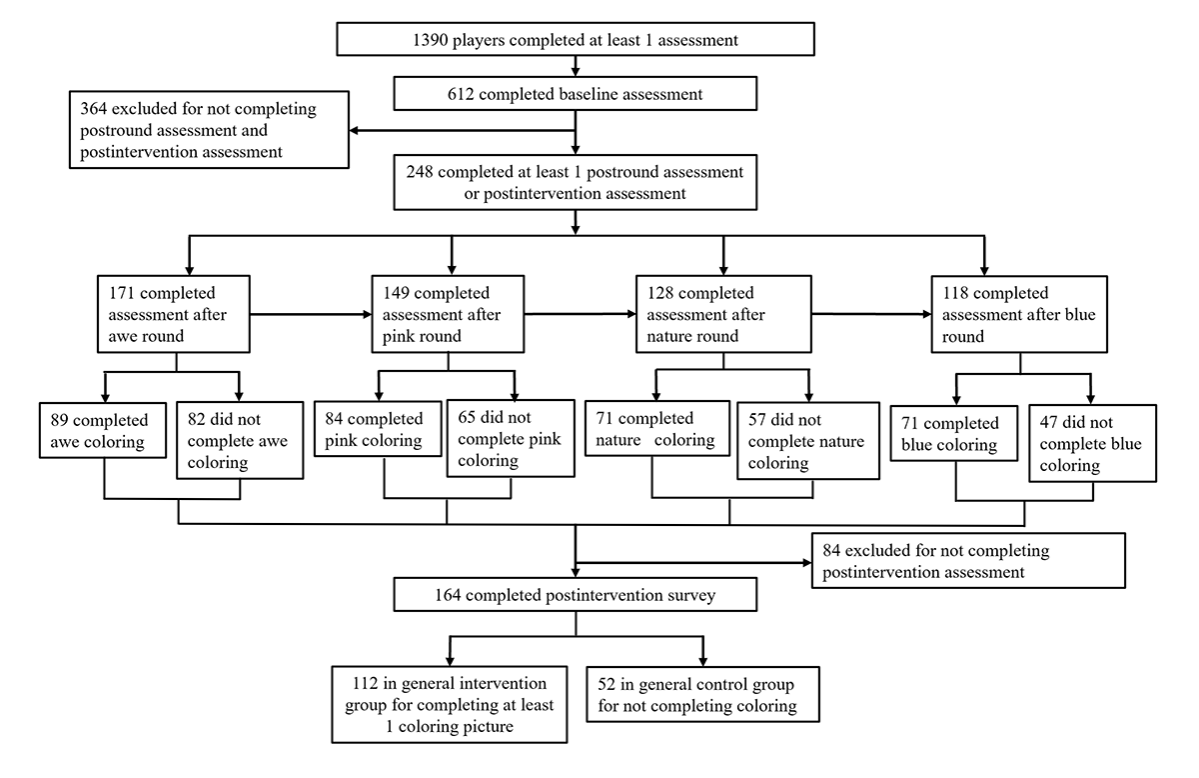
Trial profile.

In total, 1390 players from 31 countries (N_United States_=1061, 76.3%; N_United Kingdom_=124, 8.9%; N_Canada_=51, 3.7%; N_France_=33, 2.4%; N_Germany_=32, 2.3%; N_Australia_=23, 1.6%; N_others_=66, 4.7%) responded to the study and completed at least 1 assessment, of which 612 (44%) completed the baseline assessment. Of these 612 participants with baseline data, 248 (41%) completed at least 1 postround assessment or the postintervention assessment, and these participants were kept in the final sample. The participants were divided into the intervention group (colored at least 1 experimental picture in that round) and the control group (did not complete any experimental pictures in that round); see Table 1. Of the 248 participants in the final sample, 164 (66%) completed the postintervention assessment. Of these 164 participants, 112 (68%) were in the GI group because they colored at least 1 of the 16 experimental pictures and 52 (32%) were in the general control (GC) group because they did not color any experimental pictures. The study trial profile is shown in Figure 4.

The majority (n=153, 93%) of the 164 participants were over 26 years old, 146 (89%) were female, and 124 (76%) spent over 30 minutes per day playing the online game at baseline (Table 1). Players aged 26-35 years (95% CI –2.76 to –0.41, *P*=.01), 46-55 years (95% CI –2.48 to –0.22, *P*=.02), and over 56 years (95% CI –2.45 to –0.26, *P*=.02) thought the art coloring was more effective in reducing anxiety than those under 25 years old. At baseline, no significant difference was found between the GI group and the GC group in SWB (t_162_=–1.77, Cohen d=–0.29, 95% CI –0.84 to 0.05, *P*=.08), anxiety (t_162_=0.59, Cohen d=0.10, 95% CI –0.44 to 0.82, *P*=.56), SE (t_162_=1.32, Cohen d=0.22, 95% CI –0.19 to 0.93, *P*=.19), age (*χ^2^*_4_=1.89, *P*=.76), and average time spent (*χ^2^*_3_=3.00, *P*=.39).

Independent 2-tailed *t* tests were conducted to compare changes in outcome variables from baseline to postintervention in the GI and GC groups. Players' scores in the GI group showed a significantly larger increase in SWB compared to the GC group (N=164, t_162_=3.59, Cohen d=0.59, 95% CI 0.36-1.24, *P<*.001). Though no significant difference was found in change in anxiety (N=164, t_162_=–1.03, Cohen d=–0.17, 95% CI –1.11 to 0.35, *P*=.30) or change in SE (N=164, t_162_=–0.53, Cohen d=–0.09, 95% CI –0.66 to 0.39, *P*=.60) between the 2 groups (Table 2).

Statistics in specific rounds, in the awe round, 171 (69%) of the 248 players completed the baseline and postround assessment. Of these, the 89 (52%) participants in the intervention group showed a significantly higher increase in SWB than the 82 (48%) participants in the control group (N=171, t_169_=2.51, Cohen d=0.39, 95% CI 0.10-0.82, *P*=.013). Changes in anxiety (N=171, t_169_=–1.26, Cohen d=–0.19, 95% CI –0.99 to 0.22, *P*=.21) and SE (N=171, t_169_=–0.68, Cohen d=–0.11, 95% CI –0.62 to 0.30, *P*=.50) were not significantly different between the 2 groups. When the average time spent and age were taken as covariates, these conclusions were not affected.

There was an unexpected result in the pink, nature, and blue rounds. In the pink round, 149 (60%) participants completed baseline and postround assessments. Of these, 84 (56%) participants in the intervention group reported a significantly greater decrease in SWB than those in the control group (N=149, t_147_=–2.36, Cohen d=–0.40, 95% CI –0.62 to –0.05, *P*=.02). There was no significant difference in changes in anxiety (N=149, t_147_=0.33, Cohen d=0.06, 95% CI –0.43 to 0.61, *P*=.74) or changes in SE (N=149, t_147_=0.78, Cohen d=0.14, 95% CI –0.22 to 0.50, *P*=.44) between the 2 groups. In the nature round, 128 (52%) players completed baseline and postround assessments. Of these, 71 (55%) participants in the intervention group showed no significant difference between the 2 groups in changes in SWB (N=128, t_126_=1.21, Cohen d=0.22, 95% CI –0.15 to 0.61, *P*=.23) and changes in anxiety (N=128, t_126_=–1.06, Cohen d=–0.23, 95% CI –0.82 to 0.25, *P*=.29) but a significantly higher increase in changes in SE in the intervention group compared to the 57 (45%) participants in the control group (N=128, t_126_=2.08, Cohen d=0.37, 95% CI 0.02-0.80, *P*=.04). In the blue round, 118 (48%) players completed baseline and postround assessments. Of these, 71 (60%) included in the intervention group showed no significant difference in changes in SWB (N=118, t_116_=1.27, Cohen d=0.24, 95% CI –0.15 to 0.70, *P*=.21) , changes in anxiety (N=118, t_116_=–1.43, Cohen d=–0.27, 95% CI –0.97 to 0.16, *P*=.16), or changes in SE (N=118, t_116_=0.89, Cohen d=0.17, 95% CI –0.20 to 0.53, *P*=.38). It was verified that when the average time spent and age were taken as covariates, these conclusions were not affected.

As a further test of the intervention's effectiveness, we compared the participants in terms of how many rounds and how many pictures they completed. Among participants who completed both baseline and postintervention assessments (N=164), those who completed at least 1 round of intervention showed a significantly greater increase in SWB than those who did not undergo the intervention (112, 68%, vs 52, 34%, *F*_4,163_=3.52, partial ŋ^2^=0.081, *P*=.01). Moreover, compared to players who completed irrelevant pictures, those who completed 1-12 experimental pictures showed a significant increase in SWB after the intervention (N=164, *F*_4,163_=3.25, partial ŋ^2^=0.076, *P*=.01).

In the specific experimental rounds, analyses were conducted to compare participants based on the number of pictures they colored in each round. Results of univariate analysis showed that participants who colored either 1 or 4 awe pictures gained a nearly significant greater increase in SWB than those who did not color awe pictures (N=171, *F*_4,170_=2.34, partial ŋ^2^=0.053, *P*=.06). Those who completed 4 awe pictures had a significantly larger decrease in anxiety than those who completed one or did not color awe pictures (N=171, *F*_4,170_=3.39, partial ŋ^2^=0.075, *P*=.01). No association was found between the number of colored-picture numbers and outcome variables in the pink, nature, or blue round (Table 3).

**Table 1:** Baseline demographic characteristics.

Characteristic			GI^a^ group (N=112), n (%)		GC^b^ group (N=52), n (%)		Awe round (N=171), n (%)				Pink round (N=149), n (%)					Nature round (N=128), n (%)					Blue round (N=118), n (%)		
							Group 1^c^ (N=89)		Group 2^d^ (N=82)		Group 1 (N=84)		Group 2 (N=65)		Group 1 (N=71)			Group 2 (N=57)		Group 1 (N=71)			Group 2 (N=47)
**Gender**																							
	Male	10 (9)		8 (15)		7 (8)		9 (11)		10 (12)		6 (9)		5 (7)			7 (12)		9 (13)			6 (13)	
	Female	102 (91)		44 (85)		82 (92)		73 (89)		74 (88)		59 (91)		66 (93)			50 (88)		62 (87)			41 (87)	
**Age (years)**																							
	≤25	9 (8)		2 (4)		8 (9)		4 (4.9)		4 (5)		4 (6)		5 (7)			3 (5)		4 (6)			2 (4)	
	26-35	18 (16)		7 (13)		18 (20)		11 (13)		11 (13)		6 (9)		12 (17)			5 (9)		12 (17)			5 (11)	
	36-45	34 (30)		15 (29)		25 (28)		24 (29)		32 (38)		18 (28)		23 (32)			14 (25)		21 (30)			9 (19)	
	46-55	23 (21)		11 (21)		14 (16)		27 (33)		23 (27)		17 (26)		12 (17)			17 (30)		17 (24)			16 (34)	
	≥56	28 (25)		17 (33)		24 (27)		16 (20)		14 (17)		20 (31)		19 (27)			18 (32)		17 (24)			15 (32)	
**Average time spent (minutes)**																							
	≤5	0 (0)		3 (6)		3 (3)		0 (0)		2 (2)		1 (2)		1 (1)			0 (0)		1 (2)			1 (2)	
	5-15	5 (4)		5 (10)		6 (7)		6 (7)		4 (5)		5 (8)		3 (4)			5 (9)		3 (4)			2 (4)	
	16-30	19 (17)		8 (15)		19 (21)		16 (20)		14 (17)		10 (15)		12 (17)			11 (19)		13 (18)			6 (13)	
	≥31	85 (76)		39 (75)		61 (69)		60 (73)		64 (76)		49 (75)		55 (78)			41 (72)		54 (76)			38 (81)	

^a^GI: general intervention.

^b^GC: general control.

^c^Group 1: intervention group.

^d^Group2: control group.

**Table 2:** Independent 2-tailed *t* tests between residual scores of the intervention and control groups (N=164).

Outcome variable		Intervention group, mean (SD)	Control group, mean (SD)	*t* test (*df*)	*P* value	Cohen d
**Total (N=164)**						
	SWB^a^	0.24 (1.22)	–0.56 (1.53)	3.59 (162)	<.001	0.59
	Anxiety	–0.82 (2.22)	–0.44 (2.14)	–1.03 (162)	.30	–0.17
	SE^b^	–0.06 (1.49)	0.08 (1.77)	–0.53 (162)	.60	–0.09
**AWE round (N=171)**						
	SWB	0.20 (1.29)	–0.26 (1.08)	2.51 (169)	.01	0.39
	Anxiety	–0.48 (2.07)	–0.10 (1.91)	–1.26 (169)	.21	–0.19
	SE	–0.15 (1.37)	0.01 (1.67)	–0.68 (169)	.50	–0.11
**Pink round (N=149)**						
	SWB	–0.06 (0.72)	0.28 (1.02)	–2.36 (147)	.02	–0.40
	Anxiety	–0.40 (1.65)	–0.49 (1.53)	0.33 (147)	.74	0.06
	SE	0.10 (1.29)	–0.05 (0.78)	0.78 (147)	.44	0.14
**Nature round (N=128)**						
	SWB	–0.08 (1.09)	–0.32 (1.05)	1.21 (126)	.23	0.22
	Anxiety	–0.03 (1.26)	0.32 (1.79)	–1.06 (126)	.29	–0.23
	SE	0.20 (1.19)	–0.21 (0.98)	2.08 (126)	.04	0.37
**Blue round (N=118)**						
	SWB	0.46 (0.92)	0.19 (1.41)	1.27 (116)	.21	0.24
	Anxiety	–0.66 (1.43)	–0.26 (1.62)	–1.43 (116)	.16	–0.27
	SE	0.06 (1.07)	–0.11 (0.81)	0.89 (116)	.38	0.17

^a^SWB: subjective well-being.

^b^SE: subjective effectiveness (of art coloring in reducing anxiety).

**Table 3:** Univariate analysis between colored-picture numbers and outcome variables for each round.

Outcome variable		Colored-picture numbers in each round, mean (SD)						*F* test (*df*)		*P* value
		0	1	2	3	4				
**Awe round (N=171)**										
	SWB^a^	–0.26 (1.08)	0.17 (1.34)	–0.23 (1.09)	0.27 (1.19)	0.48 (1.34)	2.34 (4,170)		.06	
	Anxiety	–0.10 (1.91)	0.17 (1.85)	–0.62 (2.06)	–0.55 (0.82)	–1.57 (2.48)	3.39 (4,170)		.01	
	SE^b^	0.01 (1.67)	–0.29 (1.20)	–0.15 (0.90)	–0.36 (1.03)	0.22 (1.93)	0.59 (4,170)		.67	
**Pink round (N=149)**										
	SWB	0.28 (1.02)	–0.08 (0.68)	0.07 (0.83)	–0.22 (0.83)	0.00 (0.71)	1.55 (4,148)		.19	
	Anxiety	–0.49(1.53)	–0.38 (1.77)	–0.36 (1.50)	0.33 (1.12)	–1.08 (1.55)	1.10 (4,148)		.36	
	SE	–0.05 (0.78)	0.23 (1.40)	–0.07 (1.33)	–0.11 (1.05)	–0.08 (0.95)	0.57 (4,148)		.69	
**Nature round (N=128)**										
	SWB	–0.32 (1.05)	0.09 (1.04)	0.00 (1.00)	–0.20 (0.45)	–0.31 (1.26)	0.88 (4,127)		.48	
	Anxiety	0.32 (1.79)	0.11 (1.21)	–0.60 (0.89)	–0.20 (1.10)	0.08 (1.44)	0.55 (4,127)		.70	
	SE	–0.21 (0.98)	0.26 (1.36)	0.80 (0.45)	–0.20 (0.45)	0.08 (1.13)	1.72 (4,127)		.15	
	**Blue round (N=118)**									
	SWB	0.19(1.41)	0.33(0.84)	0.36(0.67)	1.17(1.60)	0.50(0.88)	1.10 (4,117)		.36	
	Anxiety	–0.26(1.62)	–0.53(1.47)	–0.38(1.09)	–0.88(1.86)	–0.17(0.96)	0.76 (4,117)		.56	
	SE	0.09(0.71)	–0.00(0.88)	0.38(1.02)	–0.32(1.46)	–0.08(0.58)	1.03 (4,117)		.39	

^a^SWB: subjective well-being.

^b^SE: subjective effectiveness (of art coloring in reducing anxiety).

## Discussion

### Principal Findings

This study developed an online gamification intervention based on an existing global game to reduce anxiety and promote SWB during the COVID-19 pandemic. The online gamified intervention based on the game form of coloring considered as a relaxing and natural activity can prompt participants’ mental state. The intervention packaged 4 types of pictures that have proved effective in previous research. We implemented a study to verify the effectiveness of both the online game–based method and intervention packages.

Taken together, participants in the GI group reported a significant increase in SWB compared to the GC group, even if they may be imperceptible. Previous studies have demonstrated that the repetition of coloring may produce a calming, almost trance-like effect [27], which could evoke participants' inner power and help them get out of negative thoughts and moods. The report showed visual art making is a relaxing experience for some [28]. However, the intervention effect of coloring was not significant in reducing anxiety or even decrease SWB in the GC group. Considering the increasing trend of the COVID-19 pandemic in the world, long-term and ongoing exposure to stressors related to COVID-19 may explain this finding. Coloring the 4 types of pictures and coloring itself that could reduce anxiety in peaceful times might successfully counteract the effect of stressors and prevent anxiety from increasing, rather than not being effective enough to reduce anxiety during an epidemic. The findings implied that some interventions proven effective in reducing psychological distress in prepandemic times might maintain the level of distress during the COVID-19 pandemic. Therefore, it should be noted that the study results of the online gamified coloring method for improving SWB and reducing anxiety may be underestimated during the pandemic.

In the specific intervention package, the awe-inspiring scenes were effective in increasing SWB. It is also noteworthy that those who completed all 4 awe pictures reported a significantly greater increase in SWB and a reduction in anxiety compared to the control group. It is possible that awe-inspiring scenes helped people to stay in the present moment, providing a sense of time expansion and creating positivity and satisfaction in their lives [15]. Another possible reason was that the awe experience distracted people from struggles in life, such as the chaos caused by COVID-19, and directed their attention to the light side of life [29]. People undergoing the outbreak of COVID-19, especially those going through lockdown, faced many uncertainties that could bring psychological distress [2]. Research has demonstrated that awe effectively mitigates anxiety in a stressful waiting period [14]. Therefore, the awe experience might reduce uncertainties by directing players’ attention to a “big picture,” improving the feeling of connectedness and promoting SWB during the stressful lockdown period.

Nevertheless, images that featured a lot of pink or blue or nature did not yield expected results, though they had positive reports. The approaches in peaceful times may not be effective enough to improve the mental state during an epidemic. Specifically, players who completed pink pictures reported a significant decrease in SWB than those in the control group. This was probably because too much pink color was used in the pink round. Although pink is shown to have a tranquilizing and calming effect on negative emotions [20,22], too much pink has been found to make people physically drained and emasculated [30], which in turn could reduce their SWB. This finding suggests that pictures with too much pink should be excluded from coloring materials. Those with less pink could also be examined for the intervention effect during the COVID-19 pandemic. Similarly, blue might be perceived as either calm and soothing or as cold and unfriendly. These materials of dual character might not be suitable for extreme situations of the pandemic.

Interestingly, in the nature round, the participants’ perceived effectiveness of the picture coloring did not reflect in the measure of SWB or anxiety. The participants might be influenced by the consensus on the approved effectiveness of nature pictures. Considering the challenging period all people were undergoing, coloring pictures of nature may release a positive signal for players to face difficult situations, which was also valuable during the pandemic. Another reason was likely to be rooted in the length of exposure to nature pictures. Previous studies have demonstrated that participants reported the most significant increase in self-esteem and mood after 5 minutes of exposure to nature pictures [31]. However, the majority of players in the nature round spent more than 5 minutes playing, which might lead to a reduction in the intervention effect of nature coloring. A simplified intervention using nature pictures that can be colored in around 5 minutes should be tested in future studies to examine the specific intervention effect of nature coloring. The third reason may be during the pandemic, people may gradually get used to the long-term stress caused by COVID-19 and tend to maintain their present anxiety levels. Future research is needed to examine the intervention effect of coloring on participants’ confidence in dealing with difficult situations.

### Strengths

Our research has several strengths. First, this study is the first to investigate the effect of an online coloring intervention on psychological well-being during the COVID-19 pandemic. This may give us a close view of how a coloring intervention works in a natural condition when people are undergoing a stressful period. Second, we demonstrated that the online digital coloring package improved people’s SWB during this period and in certain circumstances may have reduced anxiety. Third, compared to conventional interventions, a game-based alternative may more easily evoke people’s interest. The online format, with its accessibility, entertainment potential, and acceptability for people with barriers to seeking help, can be a practical approach to helping the general population during the pandemic. Fourth, the positive results and novel intervention approaches can stimulate more research of color and picture types as psychological intervention packages that can be delivered online so as to reduce the anxiety and other psychological distress in the general population during the pandemic.

### Limitations

This study has its limitations. First, to maintain the user experience of a commercially released game, we abandoned the plan of forcing all players (about 100,000 people per day) to participate in the study. Instead, we tried to keep the game experience as the usual, which was the goal of this study—to provide an unnoticeable psychological enhancement when dealing with the psychological threat of a major crisis. However, the cost was that we could not give full play to the advantages of the millions of players of the popular online game.

Second, to minimize the burden on participants and to get a better response rate, each outcome was assessed using 1-item measures. These measures were selected to shorten the length of the questionnaire in order to reduce the players’ burden and enlarge the size of the sample. The 1-item SWB question was assessed using the 1-item Face Scale, which has good psychometric properties. However, it is undeniable that large-scale standard scales can obtain more information.

Third, the gender distribution of the players in the coloring game was skewed. The players in the coloring game were predominantly female. Therefore, the research findings need further validation regarding the prevalence of the effects in both genders. However, the study is timely as to the immediate value of psychological interventions during the pandemic, considering that women are more prone to anxiety [32] and that screen time is negatively correlated with mental health [33].

Fourth, from the perspective of statistical calculation, the intervention effect of each round may have been underestimated, as the residual scores were calculated as the difference between each round and the one before it. The positive effects of one round might carry over into the next round, leading to underestimation of the intervention effect in the latter round.

Fifth, it is noteworthy that we considered the coloring intervention may have a significant long-term effect rather than an immediate effect on anxiety. However, online psychological intervention involves a complex superposition of psychological and cognitive mechanism, which cannot be fully demonstrated by separate experiments. Therefore, more game-based interventions and other coloring materials deserve further study, and long-term effects deserve continued attention.

### Conclusion

This study developed an entertaining, accessible, cross-regional, and effective online gamification intervention to promote SWB in a community sample of adults during the COVID-19 pandemic. The game-based intervention prompted coloring as a relaxing activity. Participants colored images that included elements that have been shown to be relaxing in previous research: an awe-inspiring scene, a nature scene, and scenes dominated by pink or blue. In general, the intervention group showed a significantly greater increase in SWB compared to the control group, and awe images proved an advantage in the online coloring approach.

Finally, the study adopted an existing released online game with high global activity as the vehicle, which eliminated the difficulties of communication and learning of new tools and, most importantly, showed the feasibility of applying existing commercial games embedded with scientific psychological interventions that can fill the gap in mental crises and services for a wider group of people during the pandemic. The result would inspire innovations to prevent the psychological problems caused by public emergencies and encourage more games, especially the most popular ones, to take more positive action for the common crises of humankind.

## Abbreviations

GC: general control
GI: general intervention
SE: subjective effectiveness
SWB: subjective well-being
