# Correction: Effect of the “Art Coloring” Online Coloring Game on Subjective Well-Being Increase and Anxiety Reduction During the COVID-19 Pandemic: Development and Evaluation

**DOI:** 10.2196/41253

**Published:** 2022-08-16

**Authors:** JuZhe Xi, YuHan Gao, Na Lyu, Zhuang She, XinYue Wang, Xin-An Zhang, XiaoYu Yu, WeiDong Ji, MengSheng Wei, WeiHui Dai, XueSheng Qian

**Affiliations:** 1 Shanghai Key Laboratory of Mental Health and Psychological Crisis Intervention, Affiliated Mental Health Center (ECNU), School of Psychology and Cognitive Science East China Normal University Shanghai China; 2 Xinhua Hospital Shanghai Jiao Tong University of Medicine Shanghai China; 3 College of Letters and Science University of California Berkeley, CA United States; 4 Antai College of Economics & Management Shanghai Jiao Tong University Shanghai China; 5 School of Management Shanghai University Shanghai China; 6 Affiliated Mental Health Center (ECNU), Shanghai Changning Mental Health Center Shanghai China; 7 School of Management Fudan University Shanghai China

In “Effect of the “Art Coloring” Online Coloring Game on Subjective Well-Being Increase and Anxiety Reduction During the COVID-19 Pandemic: Development and Evaluation” (JMIR Serious Games 2022;10(3):e37026) the authors made changes to affiliations 1 and 6:

1. In the originally published article, affiliation 1 was the following:

Shanghai Key Laboratory of Mental Health and Psychological Crisis Intervention, School of Psychology and Cognitive Science, East China Normal University, Shanghai, China

A subunit was added to affiliation 1, which now reads as follows:

Shanghai Key Laboratory of Mental Health and Psychological Crisis Intervention, Affiliated Mental Health Center (ECNU), School of Psychology and Cognitive Science, East China Normal University, Shanghai, China

2. In the originally published article, affiliation 6 was the following:

Shanghai Changning Mental Health Center, East China Normal University, Shanghai, China

A change was made to affiliation 6, which now reads as follows:

Affiliated Mental Health Center (ECNU), Shanghai Changning Mental Health Center, Shanghai, China

The correction will appear in the online version of the paper on the JMIR Publications website on August 16, 2022, together with the publication of this correction notice. Because this was made after submission to PubMed, PubMed Central, and other full-text repositories, the corrected article has also been resubmitted to those repositories.

